# Quantitative MRI Evaluation of Ferritin Overexpression in Non-Small-Cell Lung Cancer

**DOI:** 10.3390/ijms25042398

**Published:** 2024-02-18

**Authors:** Mekhla Singhania, Amira Zaher, Casey F. Pulliam, Khaliunaa Bayanbold, Charles C. Searby, Joshua D. Schoenfeld, Kranti A. Mapuskar, Melissa A. Fath, Bryan G. Allen, Douglas R. Spitz, Michael S. Petronek

**Affiliations:** 1 Department of Radiation Oncology, Division of Free Radical and Radiation Biology, University of Iowa, Iowa City, IA 52242, USA; 2 Department of Pediatrics, University of Iowa, Iowa City, IA 52242, USA; 3 Human Oncology & Pathogenesis Program, Memorial Sloan Kettering Cancer Center, New York, NY 10065, USA; schoenfj@mskcc.org

**Keywords:** ferritin, non-small-cell lung cancer, MRI, T_2_*, electron paramagnetic resonance spectroscopy

## Abstract

Cancer cells frequently present elevated intracellular iron levels, which are thought to facilitate an enhanced proliferative capacity. Targeting iron metabolism within cancer cells presents an avenue to enhance therapeutic responses, necessitating the use of non-invasive models to modulate iron manipulation to predict responses. Moreover, the ubiquitous nature of iron necessitates the development of unique, non-invasive markers of metabolic disruptions to develop more personalized approaches and enhance the clinical utility of these approaches. Ferritin, an iron storage enzyme that is often upregulated as a response to iron accumulation, plays a central role in iron metabolism and has been frequently associated with unfavorable clinical outcomes in cancer. Herein, we demonstrate the successful utility, validation, and functionality of a doxycycline-inducible ferritin heavy chain (FtH) overexpression model in H1299T non-small-cell lung cancer (NSCLC) cells. Treatment with doxycycline increased the protein expression of FtH with a corresponding decrease in labile iron in vitro and in vivo, as determined by calcein-AM staining and EPR, respectively. Moreover, a subsequent increase in TfR expression was observed. Furthermore, T_2_* MR mapping effectively detected FtH expression in our in vivo model. These results demonstrate that T_2_* relaxation times can be used to monitor changes in FtH expression in tumors with bidirectional correlations depending on the model system. Overall, this study describes the development of an FtH overexpression NSCLC model and its correlation with T_2_* mapping for potential use in patients to interrogate iron metabolic alterations and predict clinical outcomes.

## 1. Introduction

Cancer cells often rely on an increased iron-dependent phenotype, speculated to facilitate their high proliferative capacity [1]. Iron exists within a complex metabolic network that is tightly regulated in response to changing iron levels and cellular demands [2]. Intracellular iron is regulated by altering iron uptake through the transferrin receptor (TfR), export through ferroportin (Fpn-1), and storage in ferritin. In lung cancer, intracellular iron accumulation is associated with increased TfR, increased ferritin, and decreased Fpn-1 [1,3,4,5,6]. These dysregulations in iron homeostasis are believed to aid cancer cells in enhancing their labile iron pool (LIP) to maintain higher levels of available iron to meet the high proliferative demands of neoplastic transformation [2,7]. LIP is composed of weakly chelated, redox-active iron (Fe^2+^) that is available for use in various cellular processes. However, the LIP is tightly regulated to limit damaging redox chemistry such as the Fenton reaction, where Fe^2+^ reacts with hydrogen peroxide (H_2_O_2_) to generate the highly reactive hydroxyl (HO**^•^**) radical that catalyzes the oxidation of critical biological macromolecules, such as DNA [1,8]. These perturbations in iron metabolism in cancer cells thus present a viable therapeutic target that can enhance the effectiveness of cancer therapy. Consistently, it has recently been shown that LIP depletion via both ferritin heavy chain overexpression and iron chelation can induce replication stress to enhance therapeutic responses to chemoradiation in non-small-cell lung cancer (NSCLC) [9]. However, because iron is used in all cell types and is ubiquitous in nature, the personalization of iron metabolic therapies is a challenge. Thus, non-invasive metrics that can assess critical features of iron metabolism may be paramount to the successful implementation of iron-dependent therapeutic interventions.

Ferritin sits at the center of the iron metabolic network due to its role in the storage of iron contained within the LIP. Ferritin stores iron in a relatively stable Fe^3+^ form, which mitigates iron’s ability to catalyze the production of HO**^•^** [10]. Ferritin molecules are composed of two subunits: a heavy (H) chain and a light (L) chain. The H-chain has ferroxidase activity that catalyzes the oxidation of Fe^2+^ atoms during storage, while the L-chain, which lacks ferroxidase activity, assists in ferritin core formation [10]. Several clinical studies have shown higher serum ferritin levels and lower transferrin levels in lung cancer patients [5,6]. Furthermore, studies suggest that ferritin heavy chain (FtH) acts as a tumor suppressor gene, and overexpression of FtH attenuates lung cancer cell survival by inducing apoptosis through p53 [11]. Thus, the modulation of ferritin levels in vivo and the ability to non-invasively measure intra-tumoral ferritin Fe storage may provide valuable insights into the altered iron metabolic network in cancer cells as well as provide a diagnostic or prognostic approach to modify therapy.

Magnetic resonance imaging (MRI) is a non-invasive imaging technology that can generate comprehensive, three-dimensional anatomical images with superior soft-tissue contrast. It is routinely used for disease detection and treatment monitoring, providing an easily translatable method to personalize cancer therapy [8,12]. More recently, quantitative MRI has garnered interest in the oncology community to potentially gain more in-depth information regarding tumor biology. One method of quantitative MRI, T_2_* relaxation, is inversely proportional to iron content, as the increased paramagnetic material causes a shortening of the transverse relaxation time (T_2_) following the application of the radiofrequency pulse, leading to the generation of the T_2_* signal [12]. T_2_* relaxation has recently been shown to serve as a novel, non-invasive biomarker in human GBM subjects [13]. Moreover, T_2_* relaxation is an intrinsic MRI parameter that is sensitive to the iron oxidation state (Fe^2+^ vs. Fe^3+^) [14]. Because ferritin catalyzes the oxidation and storage of freely chelatable, redox-active ferrous (Fe^2+^) iron, a distinct paramagnetic shift is associated with ferritin expression. Our group previously demonstrated that T_2_^*^ relaxation times showed a direct, inverse relationship with ferritin heavy chain expression in an ex vivo soft-tissue sarcoma model [8]. Therefore, we hypothesized that the oxidation state specificity of T_2_* relaxation would allow for the non-invasive detection of altered ferritin expression in NSCLC associated with the enhanced therapy responses previously observed in [9]. This study utilized an inducible model of ferritin overexpression to interrogate the hypothesis that T_2_* mapping can serve as a reliable marker of changing ferritin expression in vivo.

## 2. Results

### 2.1. Iron Metabolism Is Dysregulated in NSCLC

**Figure 1:**
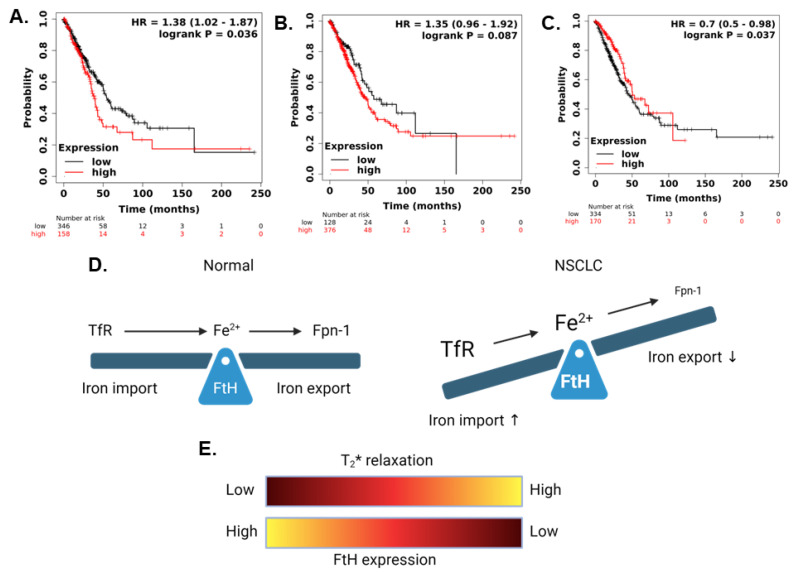
Dysregulation in iron import and export is a contributing factor to NSCLC. (**A**–**C**) Median overall survival of lung adenocarcinoma patients (*n* = 513) with differential mRNA expression of transferrin receptor (TfR, (**A**)), ferritin heavy chain (FtH, (**B**)), and ferroportin (Fpn-1, (**C**)). These data were acquired from the KM-plotter pan-cancer database (https://kmplot.com/analysis/index.php?p=service&cancer=pancancer_rnaseq; accessed on 1 September 2023). (**D**) Proposed schematic of differential iron metabolic regulation in aggressive NSCLC, where increased TfR expression and decreased Fpn-1 expression give rise to increased steady-state levels of Fe^2+^, which can facilitate tumor progression. (**E**) Schematic representation of the proposed inverse relationship between tissue FtH expression and T_2_* relaxation times. Panels (**D**,**E**) made using BioRender.com.

The disruption of normal cellular iron metabolism in cancer results in the accumulation of iron in lung cancer cells [3,4,5,6]. To understand the implications of altered iron metabolism, we assessed the expression of the following three major regulators of iron metabolism and their relationship with NSCLC clinical outcomes: transferrin receptor (TfR; TFRC), ferritin heavy chain (FtH; FTH1), and ferroportin (Fpn-1; SLC40A1). According to the KM-plotter pan-cancer RNA-seq database for lung adenocarcinomas (*n* = 513), tumors with high TfR expression correspond to a significantly worse overall survival of 39 months compared with the 54 months of the low expression cohort (HR = 1.38; 95% CI = 1.02–1.87; *p* = 0.036) (Figure 1A) [15]. High FtH expression also showed a worse prognosis, despite not reaching statistical significance (HR = 1.35; 95% CI = 0.96–1.92; *p* = 0.087) (Figure 1B). Conversely, high Fpn-1 expression was associated with a better prognosis (HR = 0.7; 95% CI = 0.5–0.98; *p* = 0.037) (Figure 1C). This is consistent with a recent report that there is increased expression of hepcidin, the negative regulator of Fpn-1, in lung adenocarcinoma that is associated with worse clinical outcomes [16]. These observations are also consistent with the hypothesis that increased Fe import, storage, and reduced export may all contribute to iron accumulation in lung cancer, contributing to aggressive cancer phenotypes [2]. Moreover, these observations suggest that highly aggressive NSCLCs present with a dysregulated iron metabolic network with increased iron uptake through TfR and decreased iron export through Fpn-1 (Figure 1D). These alterations in iron metabolism result in increased steady-state levels of labile iron, facilitating tumor growth, and identifying these targets is potentially crucial for lung cancer therapy. Consistent with this hypothesis, it has been observed that FtH overexpression promotes the induction of intrinsic apoptosis in NSCLC cells [11]. Therefore, as iron availability appears to be a critical onco-metabolite, using FtH as a regulatory model system can serve as an invaluable tool to disrupt this metabolic network to evaluate the role of iron in NSCLC progression and therapeutic responses. Moreover, because an inverse relationship has been observed between FtH expression and T_2_* relaxation (Figure 1E, [8]), this study aimed to investigate if T_2_* can be used to evaluate iron metabolic disruptions in this NSCLC model system. 

### 2.2. In Vitro Validation of Ferritin Overexpression in NSCLC Cells

**Figure 2:**
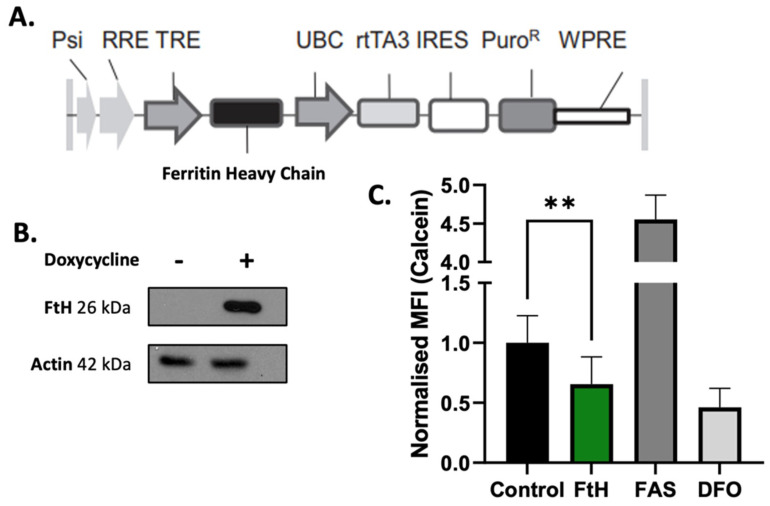
In vitro validation of ferritin overexpression in NSCLC cells. (**A**) Ferritin heavy chain (FtH)-expressing vector map. (**B**) Representative Western blot image to confirm FtH protein expression upon treatment of FtH with 1 μg mL^−1^ of doxycycline for 48 h. Actin was used as a loading control. (**C**) Changes in labile iron were measured in control and doxycycline-treated cells (1 μg mL^−1^ for 48 h) (to overexpress FtH) using a calcein-AM probe. Ferrous ammonium sulfate (FAS) (40 μM for 3 h) was used as a positive control and deferoxamine (DFO) (100 μM for 3 h) was used as a negative control. *n* ≥ 3 biological replicates with *n* ≥ 3 technical replicates per sample. Western blots are representative images of at least 3 replicates. The image of the blot was cropped to remove non-specific bands and to limit the image size. Data are represented as means ± SD. ** represents significant differences between untreated control and doxycycline-treated FtH-overexpressing cells with ** = *p* < 0.01.

To generate H1299T NSCLC cells that would conditionally overexpress FtH (H1299T FtH C11), a lentiviral doxycycline-inducible vector was constructed and utilized as described in the Section 5 (Figure 2A). The induction of H1299T FtH C11 cells with 1 µg mL^−1^ of doxycycline for 48 h led to increased protein expression of FtH in the cells (Figure 2B). To determine the effects of the overexpression of FtH on the LIP, the changes in labile iron were determined using a calcein-AM assay [17]. The results indicate that overexpression of FtH decreases LIP levels with the permeable iron chelator deferoxamine (DFO) as the negative control that decreased the LIP and ferrous ammonium sulfate (FAS) as the positive control (Figure 2C). These data show that H1299T FtH C11 cells conditionally overexpress FtH and demonstrate that FtH overexpression can deplete the LIP in vitro.

### 2.3. In Vivo Validation of H1299T FtH C11 Cells

**Figure 3:**
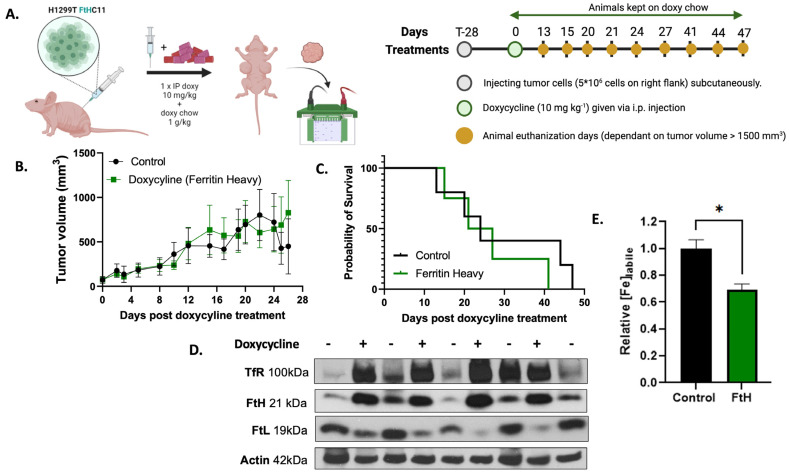
In vivo validation of ferritin overexpression in an NSCLC murine xenograft model. (**A**) Diagram depicting method of overexpressing doxycycline in murine model and timeline of treatment. Figure created using BioRender. (**B**) Tumor volume (in mm^3^) of subcutaneous tumors in mice measured at regular intervals. (**C**) Overall survival of mice with H1299T cells. Animals in FtH group were treated with an IP injection of doxycycline and kept on doxy chow as shown in (**A**). Tumor volumes and overall survival were evaluated from the time that doxycycline treatment began, approximately 28 days post-inoculation. (**D**) Protein expression of ferritin heavy chain (FtH), transferrin receptor (TfR), and ferritin light chain (FtL) in mouse xenograft tumors treated with or without doxycycline to overexpress FtH. Actin was used as a loading control. (**E**) NSCLC control and doxycycline tumor tissue were assayed for labile iron content by EPR quantification of the high-spin-state Fe^3+^-DFO complex (*g* = 4.3, 100 K). Each data point represents the average of triplicate technical replicates with * *p* < 0.05. Panel (**A**) made using BioRender.com.

Compared with the H1299 parental cell line, the H1299T FtH C11 cells demonstrated a more aggressive phenotype in nude mice, as demonstrated by the 100% tumor formation with 1 × 10^6^ cells injected versus the 12% tumor formation in the parental cell line. To determine if H1299T FtH C11 cells overexpress FtH in vivo, mice were injected with H1299T FtH C11 cells to develop subcutaneous flank tumors. Once the tumors were palpable (100 mm^3^), the mice were administered intraperitoneally with doxycycline followed by a daily doxy chow, as discussed in the Section 5 (Figure 3A). Tumor volumes were measured at regular intervals (every day or every other day), and no difference in tumor volume was observed between the two groups (Figure 3B). Once the tumor volumes reached 1500 mm^3^ (at 15–40 days), the mice were euthanized, and their tumor tissues were collected. No differences in overall survival were observed between the groups (Figure 3C). Western blot analysis was performed on tumor tissue homogenates to validate overexpression. Increases in FtH expression were observed in mice tumors that received doxycycline compared with controls (Figure 3D). Furthermore, increases in TfR expression and decreased ferritin light chain (FtL) expression were also observed. These findings suggest that LIP changes caused by FtH induce an adaptive response that is compensated for by changes in TfR and FtL expression. Finally, EPR spectroscopy was used to assess if alterations in the protein expression of FtH were associated with changes in the labile iron pool. The results indicate that FtH overexpression significantly decreases labile iron in vivo (Figure 3E). Overall, these data demonstrate that H1299 FtH tumors in mice treated with doxycycline overexpress functional FtH in vivo, which limits tumoral LIP and induces an adaptive iron metabolic response.

### 2.4. MRI T_2_* Relaxation Times Positively Correlated with Ferritin Overexpression

**Figure 4:**
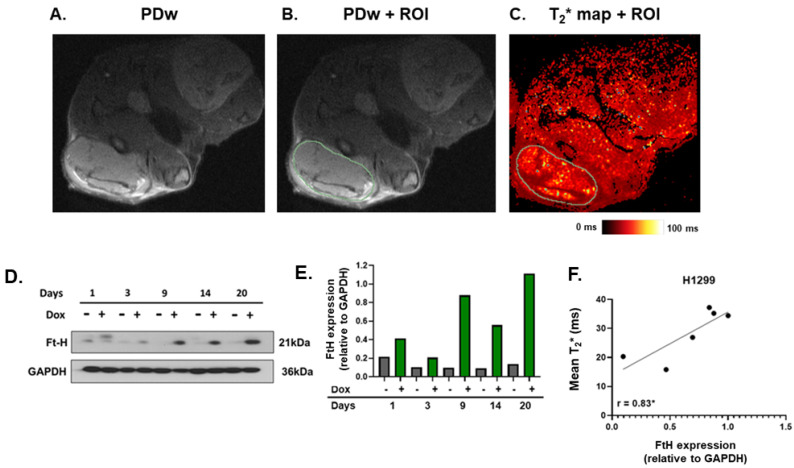
T_2_* relaxation times positively correlated with ferritin overexpression. (**A**–**C**) Representative NSCLC MRI. (**A**) Proton density-weighted MRI was used to evaluate the subcutaneous tumor structure. (**B**) Tumor region of interest (ROI—green contour) was generated on the proton density-weighted image for analysis. (**C**) Tumor ROI was applied to the associated T2* map to quantitively evaluate mean T2* relaxation times. (**D**) Western blot analysis for FtH expression in tumors homogenized following euthanasia. (**E**) Quantification of Western blot analysis shown in (**D**). FtH expression was normalized to GAPDH as an internal control. (**F**) Correlation between tumor T_2_* relaxation time and associated FtH expression using Pearson’s correlation coefficient, r, with * *p* < 0.05.

Finally, this model system was used to evaluate whether T_2_* mapping can serve as a non-invasive marker of altered FtH expression. Canonically, T_2_* mapping is considered to be a marker of total Fe content, primarily used in the evaluation of heart and liver iron overload [18,19]. However, due to the iron oxidation state specificity of T_2_* mapping, where more paramagnetic species (e.g., Fe^3+^ stored within the ferritin shell) result in shorter relaxation times, it was hypothesized that FtH overexpression would cause detectable decreases in T_2_* relaxation [14]. Consistently, human sarcoma tumors that were analyzed following surgical resection showed the expected inverse correlation with FtH expression, although this has yet to be robustly interrogated in vivo [8]. To determine if FtH overexpression could be detected in vivo using T_2_* mapping, mice were imaged immediately prior to euthanasia at days 3 and 9 to interrogate the overexpression of FtH using MRI (Figure 4A–C; see methods for details), while tissues harvested at other time points were utilized for model validation via Western blotting. FtH overexpression was confirmed in vivo, supporting the reproducibility of this model system (Figure 4D). The protein levels at each time point were quantified relative to a GAPDH loading control (Figure 4E). Tumor regions were defined as hyperintense regions in the proton-density-weighted MRI, with the contour then being used for T_2_* analysis. The T_2_* values determined prior to euthanasia strongly correlated with FtH expression (Figure 4F; r = 0.83, *p* < 0.05). However, the evaluated tumor regions exhibited a high level of intra-tumoral variability, as evidenced by a mean standard deviation of ±29.4 ms (Supplementary Table S1). This is consistent with the general heterogeneity of tumor tissue. Therefore, these data suggest the reliability of the in vivo model of FtH overexpression and that alterations in the LIP levels can be monitored in vivo using T_2_* mapping.

**Figure 5:**
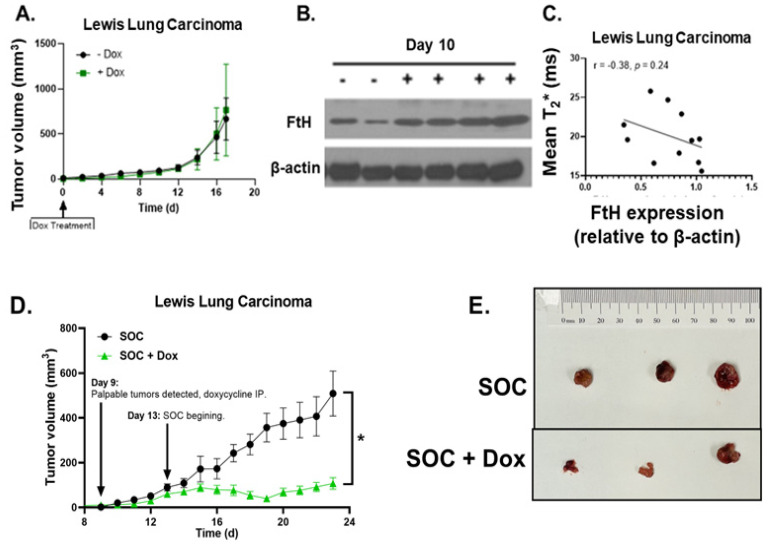
T_2_* relaxation times inversely correlated with FtH expression in an immune-competent syngeneic model system. (**A**) Tumor growth rates for Lewis lung carcinoma (LLC) tumors with and without doxy-inducible FtH overexpression (*n* = 3 animals, with two bilateral tumors per animal). Animals were given an IP injection of 10 mg/kg of doxycycline and continuous 1 g/kg of doxycycline feed once tumors were palpable (time 0, *y*-axis). (**B**) Representative Western blot of FtH expression at day 10 to validate FtH overexpression in LLC tumors. (**C**) Negative correlation between relative FtH protein expression and T_2_* relaxation time in images collected on day 10 and day 17. (**D**) Lewis lung carcinoma tumor growth following treatment with standard of care (SOC, *n* = 5) ± FtH overexpression (SOC + Dox, *n* = 5). * *p* < 0.05. (**E**). Representative images showing Lewis lung carcinoma tumors at the end of two weeks of SOC treatment with and without FtH overexpression.

For further model validation, this experiment was repeated in a second mouse model system of NSCLC by injecting doxycycline-inducible FtH-overexpressing Lewis lung carcinoma (LLC FtH C3) cells into immune-competent C57BL6/J mice. Consistent with our preliminary validation studies, the use of doxycycline showed no effect on the tumor growth rates (Figure 5A). Tumor tissues were harvested at day 10 to validate FtH overexpression in this model system to further validate that overexpression of FtH was able to be replicated in vivo (Figure 5B). While mice were euthanized to confirm overexpression throughout the duration of this study, the mice in both the doxycycline and control groups (*n* = 11 total) were scanned immediately prior to euthanasia at days 10 and 17, as previously performed, to evaluate the T_2_* data. In this experiment, we observed an inverse correlation between the T_2_* relaxation time and FtH expression (Figure 5C). A high level of tissue heterogeneity was also observed in this model system (mean standard deviation = ±23.46 ms; Supplementary Table S1).

Lastly, we aimed to replicate our previous results demonstrating that FtH overexpression in H1299T cells enhances therapeutic responses to chemoradiation [9]. Consistent with our previous results in human xenografts, we observed that FtH overexpression significantly delayed tumor growth in syngeneic Lewis lung carcinoma cells (Figure 5D,E). In total, it appears that T_2_* mapping can detect FtH expression in vivo, but the relationship appears to be model-system-dependent. Importantly, LIP depletion appears to remain an effective therapeutic strategy for the management of NSCLC. 

## 3. Discussion

### 3.1. Model Validation

The overarching goal of this study was to validate doxycycline-inducible FtH overexpression in an NSCLC model (H1299T FtH C11) and use this model system to evaluate the ability of T_2_* mapping to detect changes in FtH expression in vivo above the noise of other factors, such as H_2_O/fluid accumulation or necrosis [20]. We observed that FtH was overexpressed in vitro and in vivo in our model systems (Figure 2 and Figure 3). Notably, while FtH was overexpressed at the protein level, it was also overexpressed as a functional enzyme. This was evident in the overexpression of FtH leading to subsequent decreases in labile iron both in vitro and in vivo. These results are consistent with the functionality of FtH as the primary iron storage enzyme [6]. Moreover, the decrease in labile iron upon the overexpression of FtH induced an iron metabolic shift in vivo, further highlighting the biological relevance of this model system. Iron is required by a variety of cellular processes, such as the incorporation of iron into iron–sulfur clusters required for high-fidelity DNA replication [7]. Therefore, the removal of intracellular LIP via FtH overexpression may allow for a greater understanding of the role of iron in tumor development and progression. This has been evidenced by recent data indicating that LIP depletion induces replication stress to enhance responses to chemoradiation [21], an effect that we were able to replicate in an immune-competent model system using Lewis lung carcinoma cells (Figure 5D,E). Overall, we successfully generated two fully functional, biologically relevant FtH overexpression model systems in NSCLC, which may interest the cancer biology community as iron accumulation and altered iron metabolism have been reported to promote NSCLC incidence and progression [2,3,17,22]. This model may also be extended beyond NSCLC to evaluate iron metabolic alterations, as iron is believed to be central to cancer development and progression in a variety of tumors [2]. 

### 3.2. Implications of T_2_* Detection of Ferritin Expression

In addition to initial development and validation, this model was evaluated using T_2_* mapping. Previously, it has been reported that T_2_* mapping can detect FtH expression in soft-tissue sarcomas [8]. Herein, we observed bidirectional correlations between FtH expression and T_2_* relaxation. This result is somewhat non-canonical because ferritin is a large, paramagnetic molecule that tends to cause a decrease in T_2_* relaxation times [10]. These non-canonical results were observed in two different model systems, including both human tumor xenografts as well as a syngeneic mouse model system of NSCLC. Thus, it appears that T_2_* mapping may be employed while using model systems for the in vivo expression tracking of FtH, which is highly context-dependent. There are multiple possible explanations for the differential correlations that were observed in this study that require further investigation. Based on the observed changes in FtL and TfR expression, it is evident that the chronic overexpression of FtH induces iron metabolic changes. Because T_2_* relaxation times are traditionally inversely correlated with the total iron content and can be inversely correlated with FtH [8,23], the observed positive correlation may be related to changes in iron efflux as the modulation of ferroportin expression with hepcidin has been shown to alter T_2_* relaxation times [24]. Moreover, the differential correlations may also be related to the recycling of ferritin, as there may be a tumor cell-specific activation of ferritinophagy. Thus, it may be pertinent to investigate the relationship between T_2_* relaxation and ferritin-specific iron metabolic enzymes, such as NCOA4 (in ferritinophagy and ferritin degradation [25]) and PROM2 (in ferritin efflux [26]). While these data may signify a potential study limitation, these data may also suggest that T_2_* mapping may have the potential to provide deeper iron metabolic information regarding the use and recycling of ferritin, which warrants further investigation. 

### 3.3. Study Limitations: Technical Challenges and Future Perspectives

Moreover, the utilization of a subcutaneous model system presents a technical limitation for using T_2_* mapping in NSCLC, which requires further investigation and developmental effort. This is because the anatomical environment used herein (i.e., a subcutaneous flank model) may not reflect a true human in vivo study, since NSCLC tumors arise in the lungs that typically have a large air–tissue interface. This air–tissue interface of the lungs can cause significant susceptibility artifacts that require technical interventions to diminish this effect [27]. Thus, the application of T_2_* appraised in this article will require further technical developments, such as the use of ultrashort-echo-time T_2_* mapping [21], and should be investigated in orthotopic models and clinical settings. Ultrashort-echo-time T_2_* mapping has previously been shown to be a reasonable approach to evaluate the short T_2_* relaxation times of the lung parenchyma (0.5–3 ms) [28]. Another potential challenge with this approach is the high intra-tumoral variability in the T_2_* relaxation times within tumors, as observed in the large standard deviations in this study. However, tumors are notoriously heterogeneous. which can be exacerbated following some type of biochemical modification (e.g., radiotherapy) [29]. Thus, tumor T_2_* heterogeneity may also provide valuable information, as increased apparent diffusion coefficient standard deviations positively correlate with the endometrial cancer grading [30]. Therefore, while the tumor standard deviations appear high in this context, they may also provide valuable information moving forward. Moreover, the use of a 7T magnet may also limit the utility of this study as a 3T magnetic is used clinically; however, a recent study on glioblastoma subjects showed that at 3T, T_2_* mapping is a reliable marker of treatment responses [13]. These clinical responses directly correlated with pre-clinical validation using a 7T magnet, supporting the future translational capacity of this approach. Therefore, despite the preliminary nature of this work, the use of T_2_* mapping in NSCLC has translational promise that warrants further interrogation. 

## 4. Conclusions

This study reports the preliminary development and validation of a FtH overexpression model in NSCLC. Importantly, this model system allows the in vivo expression of the functional FtH enzyme that sequesters labile iron to induce iron metabolic changes. Additionally, we observed that T_2_* mapping can detect FtH expression in this model system to serve as an in vivo monitor of iron metabolism. Moreover, using two different NSCLC models, we found that T_2_* has the potential to detect FtH expression in vivo and the differential correlations observed may be related to more intricate iron metabolic mechanisms associated with the long-term overexpression of FtH. Therefore, T_2_* appears to have the potential to evaluate iron metabolic changes associated with FtH expression, which warrants further consideration.

## 5. Methods 

### 5.1. Cell Culture and Ferritin Overexpression Development

H1299 and Lewis lung carcinoma cells were obtained from ATCC (Gaithersburg, VA, USA) (CRL-5803 and CRL-1642, respectively). To produce a more aggressively growing variant of H1299 for use in xenograft model systems, 1 × 10^6^ H1299 cells were injected subcutaneously in the right rear flank of female 4–6-week-old athymic nude mice (Foxn1nu/Foxn1nu) purchased from Envigo (previously, Harlan Laboratories, Indianapolis, IN, USA). Tumors that reached 1000 mm^3^ were removed, washed in sterile PBS, minced in DMEM + 10% fetal bovine serum (FBS) + penicillin/streptomycin (PS) containing collagenase/hyaluronidase (Stemcell–#07912) and DNase (Roche 11284932001), and incubated overnight at 37 °C in a 5% CO_2_ incubator. The supernatant with digested cells was aspirated and transferred to a 60 mm dish in fresh complete medium (DMEM + 10% FBS + PS) and incubated at 37 °C with a medium change after 24 h. The cells were allowed to grow to confluence, and the cells were passaged 5 more times every 3–4 days to allow any fibroblasts to be depleted from the population. The cells were then renamed H1299T to indicate the passage of H1299 as a tumor through the mice prior to re-isolation. The H1299T cells were then confirmed by the ATCC Cell Line Authentication Service STR report, showing a 92% match with the database profile for H1299, meeting the criteria for being derived from this parental cell line. We then determined that the H1299T cells were mycoplasma-negative and froze down multiple aliquots in liquid nitrogen and −80 °C freezers for future experimentation.

Cells were grown in RPMI medium with 10% fetal bovine serum (FBS) in an incubator at 37 °C, 5% CO_2_, and ambient O_2_. All cell lines were utilized before passage 15 and treated in an exponential growth phase at 70–80% confluence. Briefly, ferritin heavy chain 1 (FtH)-overexpressing cell lines were constructed by PCR amplification from a human liver cDNA library (Clontech, Mountain View, CA, USA), and the PCR product and pTRIPZ vector were cut with AgeI and MluI and ligated using T4 DNA ligase and then transformed into STLB3 competent cells from Invitrogen, and the plasmid was extracted and sequenced. The FtH plasmid was transfected into 293FT cells using the helper vectors psPAX2 and pCMV-VSV-G (Addgene, Watertown, MA, USA) to produce lentivirus, which was then used to transduce the H1299T and Lewis lung carcinoma (LLC) cells. The cells were plated and allowed to grow for 48 h, and then the virus was added to the cells with 8 µg/mL of polybrene for a total of 48 h, with fresh virus being added after 24 h. After transduction, cells were selected with puromycin (6 μg/mL). For the expansion of single transduced clones, cells were re-plated in 150 mm dishes, with 1000 cells per plate, and were grown for 10 days before 12 clones were selected for expansion. Following expansion, the selected clones were tested for FtH overexpression in vitro after treatment with doxycycline (1 µg/mL) for 24, 48, and 72 h, and clones 11 (H1299T FtH C11) and 3 (LLC FtH C3) were used for further experimentation. 

### 5.2. Western Blot Analysis 

The protein concentrations in samples were determined by Lowry protein assay using the Bio-rad DC protein assay kit. Following this, the samples were lysed in a lysis buffer (RIPA). The samples were run on a 4–20% gradient gel at 115 V for 75 min. The proteins were then transferred from the gel to the membrane. To check if all proteins had been transferred, Ponceau Red staining of the membrane and Coomassie Blue staining of the gel were performed. To block non-specific protein binding, the membrane was incubated for 1 h in a buffer containing 5% non-fat dry milk. Following this, the membrane was incubated with an anti-ferritin heavy chain antibody (FtH) (1:1000, 77127, Abcam, Waltham MA, USA), anti-transferrin receptor antibody (TfR-1) (1:2000, 136800, Life Technology, Carlsbad, CA, USA), and anti-ferritin light chain antibody (FtL) (1:1000, 109373, Abcam, Waltham MA, USA). After 3 × 5 min PBS-Tween washes, the membranes were incubated with an anti-mouse secondary antibody (1:20,000; Sigma-Aldrich, St. Louis, MO, USA). Finally, the washed membranes were incubated with Super Signal West Pico Chemiluminescent Substrate (ThermoScientific, Waltham, MA, USA) and exposed to CareStream BioMax MR Film (CareStream Health, Rochester, NY, USA) to visualize the corresponding bands. 

### 5.3. Animal Studies

All procedures were approved by the University of Iowa IACUC (#2031774). The animals were housed at the University of Iowa Animal Care Facility in a temperature-controlled room with a 12 h dark/light cycle. The mice were maintained on normal diets (with a special diet during the doxycycline induction phase, as detailed below) and water ad libitum throughout the course of the experiment. 

#### 5.3.1. Immune-Deficient Model

Female 4–6-week-old athymic nude mice were injected subcutaneously with 5 × 10^6^ H1299T cells with doxycycline-inducible FtH overexpression in the rear right flank.

#### 5.3.2. Immune-Competent Model

Female 4–6-week-old C57BL/6J mice (Charles River, Wilmington, MA, USA) were injected subcutaneously with 1 × 10^6^ Lewis lung carcinoma cells (LLCs) with doxycycline-inducible FtH overexpression in the rear right flank. The standard of care (SOC) with carboplatin/paclitaxel + ionizing radiation was administered for two weeks as follows: carboplatin (5 mg kg ^−1^)/paclitaxel (20 mg kg^−1^) were delivered once per week, intraperitonially, and radiation was delivered as 4 fractions × 2 Gy (8 Gy total) to the flank tumors (2 fractions per week) using an Xstrahl Small Animal Radiation Research Platform (SARRP). 

#### 5.3.3. Procedure

Following the formation of palpable tumors, animals were randomly assigned to experimental groups including control and doxycycline overexpression groups. Once the tumors reached 100 mm^3^ in size, the mice in the doxycycline group were treated with a single dose of doxycycline given intraperitoneally (10 mg kg^−1^). The control mice were treated with an equivalent dose of NaCl. The mice in the doxycycline group were fed with chow (Envigo TD.05298, Indianapolis, IN, USA) infused with 1 g kg^−1^ of doxycycline for the duration of the experiment. Tumors were measured every day, or every other day, with Vernier calipers (volume = (length × width × (width/2)), and the mice were euthanized when the tumor length exceeded 1.5 cm in any dimension for two consecutive days. Mice were terminally anesthetized using 200 µL of intraperitoneal injection of a ketamine/xylazine 17.5/2.5 µg/µL mix. When analgesia was achieved, as determined by a lack of the toe pinch response, cervical dislocation was followed by cardiac puncture to withdraw blood. Tumor tissue was flash-frozen in liquid nitrogen and stored in a −80 °C freezer for further analysis.

### 5.4. Labile Iron Measurement in Cultured Cells

The labile iron pool (LIP) in cultured cells was determined as previously described in [9]. Briefly, after sample treatment, the cells were trypsinized, centrifuged at 1200 rpm for 5 min, and resuspended at approximately 1 × 10^6^ cells mL^−1^ in 500 nM of calcein-AM in PBS. The samples were incubated for 15 min at 37 °C. Subsequently, the samples were pelleted, washed in PBS, and resuspended in 1 mL of PBS before dividing each sample into two flow cytometry sample tubes, where 100 mM of 2′,2′-bipyridyl (BIP) was added to one of these tubes. The samples were kept at room temperature, and 10,000 cells were analyzed with an LSR II Flow Cytometer (BD Biosciences, Franklin Lakes, NJ, USA; ex = 488 nm, em = 515/20 nm). The BIP tubes were incubated for at least 15 min before analysis to allow for the full chelation of intracellular labile iron. The LIP (A.U.) = MFI BIP—MFI No BIP was normalized against the control samples to calculate the relative labile iron pool.

### 5.5. EPR Measurements

Following the tissue collection, the tumors were flash-frozen with liquid nitrogen and stored at −80 °C until the sample preparation. The samples were homogenized using 2 µL per mg tissue of phosphate-buffered saline (pH = 6.5) containing 5 mM of deferoxamine (deferoxamine mesylate, DFO; D9533, Sigma-Aldrich, St. Louis, MO, USA). The samples were incubated on ice for a minimum of 2 h in the presence of oxygen to allow the DFO to complex the freely chelatable iron and ensure subsequent oxidation to form the ferrioxamine complex (DFO-Fe^3+^). Labile iron concentrations were evaluated by monitoring the high-spin Fe^3+^ ferrioxamine (DFO-Fe^3+^) complex at *g* = 4.3 (T ≈ 200 K) using a Bruker EMX electron paramagnetic resonance spectrometer (Bruker ER4111VT variable temperature accessory and standard TE cavity, Billerica, MA, USA), as previously described in [31]. The mean signal intensity (A.U.) from triplicate measurements was used to determine the labile iron concentration. 

### 5.6. MRI Parameters 

The mice were temporarily anesthetized using isoflurane, and T_2_*-weighted images of their subcutaneous tumors were collected using a gradient echo sequence (TR = 10 ms; TEs = 2.2, 8.2, 14.2, and 20.2 ms; field of view = 2.0 × 2.0 cm; matrix = 256 × 256; signal averages = 2) with a 7T GE small animal scanner, a part of the Small Animal Imaging Core at the University of Iowa. Tumor images were generated by having the mouse lie flat and placing the subcutaneous flank tumor directly against a ^1^H 300 MHz rat head coil (RAPID Biomedical GmbH; Rimpar, Germany). Prior to the collection of gradient echo images, a 4 cm higher-order shim was used to minimize B_0_-field inhomogeneities. T_2_* maps were generated by fitting each voxel to a mono-exponential curve across the four echoes acquired using an in-house Python code. The images were imported to Slicer3D software V5.0.3, where the regions of interest (ROIs) were delineated on a proton density-weighted image and applied to the associated T_2_* map for analysis (Figure 4A–C). Mean T_2_* values were calculated using the label statistics tool within Slicer3D [8]. For quality assurance, increasing concentrations of Fe(NO_3_)_3_ embedded in 1% agarose gel (20–80 µM) were evaluated using this gradient-echo imaging protocol to determine our ability to reliably detect changes in iron concentrations. A linear relationship was observed between T_2_* relaxation and [Fe(NO_3_)_3_] (R^2^ = 0.8), with a slope of 0.16 ± 0.03 µM ms^−1^. 

## Data Availability

Data is available at the request of the corresponding author(s).

## Supplementary Materials

The following supporting information can be downloaded at https://www.mdpi.com/article/10.3390/ijms25042398/s1.

## References

[1] Torti S.V., Torti F.M. (2013). Iron and Cancer: More Ore to Be Mined. Nat. Rev. Cancer.

[2] Petronek M.S., Spitz D.R., Buettner G.R., Allen B.G. (2019). Linking Cancer Metabolic Dysfunction and Genetic Instability through the Lens of Iron Metabolism. Cancers.

[3] Sukiennicki G.M., Marciniak W., Muszyńska M., Baszuk P., Gupta S., Białkowska K., Jaworska-Bieniek K., Durda K., Lener M., Pietrzak S. (2019). Iron Levels, Genes Involved in Iron Metabolism and Antioxidative Processes and Lung Cancer Incidence. PLoS ONE.

[4] Alemán M.R., Santolaria F., Batista N., de La Vega M.J., González-Reimers E., Milena A., Llanos M., Luis Gómez-Sirvent J. (2002). Leptin Role in Advanced Lung Cancer. A Mediator of the Acute Phase Response or a Marker of the Status of Nutrition?. Cytokine.

[5] Ferrigno D., Buccheri G. (1992). Serum Ferritin Levels in Lung Cancer Patients. Eur. J. Cancer.

[6] Yildirim A., Meral M., Kaynar H., Polat H., Ucar E.Y. (2007). Relationship between Serum Levels of Some Acute-Phase Proteins and Stage of Disease and Performance Status in Patients with Lung Cancer. Med. Sci. Monit..

[7] Petronek M.S., Spitz D.R., Allen B.G. (2021). Iron–Sulfur Cluster Biogenesis as a Critical Target in Cancer. Antioxidants.

[8] Petronek M.S., Tomanek-Chalkley A.M., Monga V., Milhem M.M., Miller B.J., Magnotta V.A., Allen B.G. (2022). Detection of Ferritin Expression in Soft Tissue Sarcomas With MRI: Potential Implications for Iron Metabolic Therapy. Iowa Orthop. J..

[9] Bayanbold K., Singhania M., Fath M.A., Searby C.C., Stolwijk J.M., Henrich J.B., Pulliam C.F., Schoenfeld J.D., Mapuskar K.A., Sho S. (2023). Depletion of Labile Iron Induces Replication Stress and Enhances Responses to Chemoradiation in Non-Small-Cell Lung Cancer. Antioxidants.

[10] Harrison P.M., Arosio P. (1996). The Ferritins: Molecular Properties, Iron Storage Function and Cellular Regulation. Biochim. Biophys. Acta (BBA)-Bioenerg..

[11] Biamonte F., Battaglia A.M., Zolea F., Oliveira D.M., Aversa I., Santamaria G., Giovannone E.D., Rocco G., Viglietto G., Costanzo F. (2018). Ferritin Heavy Subunit Enhances Apoptosis of Non-Small Cell Lung Cancer Cells through Modulation of miR-125b/P53 Axis. Cell Death Dis..

[12] Chavhan G.B., Babyn P.S., Thomas B., Shroff M.M., Haacke E.M. (2009). Principles, Techniques, and Applications of T_2_*-Based MR Imaging and Its Special Applications. RadioGraphics.

[13] Petronek M.S., Monga V., Bodeker K.L., Kwofie M., Lee C.-Y., Mapuskar K.A., Stolwijk J.M., Zaher A., Wagner B.A., Smith M.C. (2023). Magnetic Resonance Imaging of Iron Metabolism with T2* Mapping Predicts an Enhanced Clinical Response to Pharmacologic Ascorbate in Patients with GBM. Clin. Cancer Res..

[14] Petronek M.S., St-Aubin J.J., Lee C.Y., Spitz D.R., Gillan E.G., Allen B.G., Magnotta V.A. (2021). Quantum Chemical Insight into the Effects of the Local Electron Environment on T2*-Based MRI. Sci. Rep..

[15] Nagy Á., Munkácsy G., Győrffy B. (2021). Pancancer Survival Analysis of Cancer Hallmark Genes. Sci. Rep..

[16] Fan Y., Liu B., Chen F., Song Z., Han B., Meng Y., Hou J., Cao P., Chang Y., Tan K. (2021). Hepcidin Upregulation in Lung Cancer: A Potential Therapeutic Target Associated With Immune Infiltration. Front. Immunol..

[17] Thielmann C.M., Costa da Silva M., Muley T., Meister M., Herpel E., Muckenthaler M.U. (2019). Iron Accumulation in Tumor-Associated Macrophages Marks an Improved Overall Survival in Patients with Lung Adenocarcinoma. Sci. Rep..

[18] Westwood M., Anderson L.J., Firmin D.N., Gatehouse P.D., Charrier C.C., Wonke B., Pennell D.J. (2003). A Single Breath-Hold Multiecho T2* Cardiovascular Magnetic Resonance Technique for Diagnosis of Myocardial Iron Overload. J. Magn. Reson. Imaging.

[19] Galimberti S., Trombini P., Bernasconi D.P., Redaelli I., Pelucchi S., Bovo G., Di Gennaro F., Zucchini N., Paruccini N., Piperno A. (2015). Simultaneous Liver Iron and Fat Measures by Magnetic Resonance Imaging in Patients with Hyperferritinemia. Scand. J. Gastroenterol..

[20] Lee C.-Y., Petronek M.S., Monga V., Miller B.J., Milhem M.M., Magnotta V.A., Allen B.G. (2023). T2* Imaging Assessment of Neoadjuvant Radiation Therapy Combined With Pharmacological Ascorbate in Extremity Soft-Tissue Sarcomas: A Pilot Study. Iowa Orthop. J..

[21] Yu J., Xue Y., Song H.K. (2011). Comparison of Lung T2* During Free-Breathing at 1.5T and 3.0T with Ultrashort Echo Time (UTE) Imaging. Magn. Reson. Med..

[22] Wang B., Zhang J., Song F., Tian M., Shi B., Jiang H., Xu W., Wang H., Zhou M., Pan X. (2016). EGFR Regulates Iron Homeostasis to Promote Cancer Growth through Redistribution of Transferrin Receptor 1. Cancer Lett..

[23] Zamani F., Razmjou S., Akhlaghpoor S., Eslami S.-M., Azarkeivan A., Amiri A. (2011). T2* Magnetic Resonance Imaging of the Liver in Thalassemic Patients in Iran. World J. Gastroenterol..

[24] Alizadeh K., Sun Q., McGuire T., Thompson T., Prato F.S., Koropatnick J., Gelman N., Goldhawk D.E. (2020). Hepcidin-Mediated Iron Regulation in P19 Cells Is Detectable by Magnetic Resonance Imaging. Sci. Rep..

[25] Mancias J.D., Wang X., Gygi S.P., Harper J.W., Kimmelman A.C. (2014). Quantitative Proteomics Identifies NCOA4 as the Cargo Receptor Mediating Ferritinophagy. Nature.

[26] Brown C.W., Amante J.J., Chhoy P., Elaimy A.L., Liu H., Zhu L.J., Baer C.E., Dixon S.J., Mercurio A.M. (2019). Prominin2 Drives Ferroptosis Resistance by Stimulating Multivesicular Body/Exosome-Mediated Iron Export. Dev. Cell.

[27] Vignaud A., Maître X., Guillot G., Durand E., de Rochefort L., Robert P., Vivès V., Santus R., Darrasse L. (2005). Magnetic Susceptibility Matching at the Air–Tissue Interface in Rat Lung by Using a Superparamagnetic Intravascular Contrast Agent: Influence on Transverse Relaxation Time of Hyperpolarized Helium-3. Magn. Reson. Med..

[28] Bergin C.J., Pauly J.M., Macovski A. (1991). Lung Parenchyma: Projection Reconstruction MR Imaging. Radiology.

[29] Tomaszewski M.R., Dominguez-Viqueira W., Ortiz A., Shi Y., Costello J.R., Enderling H., Rosenberg S.A., Gillies R.J. (2021). Heterogeneity Analysis of MRI T2 Maps for Measurement of Early Tumor Response to Radiotherapy. NMR Biomed..

[30] Yan B., Liang X., Zhao T., Ding C., Zhang M. (2020). Is the Standard Deviation of the Apparent Diffusion Coefficient a Potential Tool for the Preoperative Prediction of Tumor Grade in Endometrial Cancer?. Acta Radiol..

[31] Moser J.C., Rawal M., Wagner B.A., Du J., Cullen J.J., Buettner G.R. (2014). Pharmacological Ascorbate and Ionizing Radiation (IR) Increase Labile Iron in Pancreatic Cancer. Redox Biol..

