# Supplementary material for: Quantitative MRI Evaluation of Ferritin Overexpression in Non-Small-Cell Lung Cancer
Source: Int J Mol Sci. 2024 Feb 18;25(4):2398. doi: 10.3390/ijms25042398 (PMC10889593; doi:10.3390/ijms25042398)
Supplement: Supplementary file 1 [file ijms-25-02398-s001.zip › ijms-2854073-supplementary.pdf]

**Table S1.** *T2* values correlated with FtH expression used in this study.*				
	**T2* (ms)**	**SD**	**$^{a}R^2$**	Doxycycline
**H1299T**				
**1**	20.3	26.8	0.87	-
**2**	26.9	15.8	0.95	-
**3**	35.2	45.9	0.96	+
**4**	37.2	42.1	0.92	+
**5**	15.8	6.1	0.97	+
**6**	34.4	39.9	0.91	+
**Mean**	28.3	29.4	0.93	
**Lewis Lung Carcinoma**				
**1**	19.6	17.8	0.92	-
**2**	21.5	26.2	0.9	-
**3**	22.9	28.6	0.87	+
**4**	17.9	12.7	0.96	+
**5**	16.6	25.7	0.9	+
**6**	19.7	28.5	0.9	+
**7**	24.7	32.9	0.88	+
**8**	25.8	29.1	0.93	+
**9**	21.5	26.2	0.9	+
**10**	16.7	15.9	0.92	+
**11**	19.5	17.2	0.92	+
**Mean**	20.5	23.46	0.91	

$^{a}R^2$ values were generated for each voxel to evaluate the quality of the T_2* fitting.
